# Advances and prospects of ergothioneine in the treatment of cognitive frailty

**DOI:** 10.1080/07853890.2025.2555742

**Published:** 2025-09-07

**Authors:** Arjun M. M. I. Gede, Qingxin Gu, Piyaporn Phukhatmuen, Jian Xiong, Shuang Zhang, Mingliang Yi, Sitthichock Vadphimai, Wenchuan Qi

**Affiliations:** a School of Acupuncture–Moxibustion and Tuina, Chengdu University of Traditional Chinese Medicine, Chengdu, China; b School of Integrative Medicine, Mae Fah Luang University, Chiang Rai, Thailand; c Ministry of Education, Key Laboratory of Acupuncture for Senile Disease (Chengdu University of TCM), Chengdu, China

**Keywords:** Antioxidant, clinical trials, cognitive frailty, ergothioneine, neuroprotective

## Abstract

**Background:**

To review the biological functions of ergothioneine (ERGO), its correlation with plasma levels in cognitive frailty, and research progress in treating frailty and cognitive impairment, with the aim of providing a reference for ERGO application in cognitive frailty treatment.

**Methods:**

A comprehensive review of existing literature on ERGO’s chemical structure, sources, antioxidant and anti-inflammatory effects, and its role in cognitive frailty was conducted. Clinical trial data and metabolomic studies were also analyzed to understand ERGO’s therapeutic potential.

**Results:**

ERGO, a naturally occurring antioxidant, exhibits strong antioxidant, anti-inflammatory, and immunomodulatory activities. Age-related declines in plasma ERGO levels are observed, particularly in individuals with cognitive impairment. Metabolomic analyses confirm ERGO’s beneficial effects on cognition and memory. Preclinical studies demonstrate ERGO’s capacity to enhance cognitive function and neuronal health through oxidative stress reduction and neuroprotection.

**Conclusion:**

Preclinical studies have underscored ERGO’s potent antioxidant, anti-inflammatory, and neuroprotective effects, suggesting its potential as a therapeutic agent for cognitive frailty. However, the translation of these findings into clinical benefits necessitates validation through well-designed clinical trials. While existing evidence is promising, suggesting ERGO as a viable complementary intervention, comprehensive and rigorous studies are imperative to establish its clinical efficacy and safety in managing cognitive frailty.

## 1. Introduction

Cognitive frailty, first formally proposed by the International Society of Nutrition and Aging and the International Geriatrics and Gerontology Association in 2013, is a clinical state where physical frailty and cognitive impairment coexist. The cognitive impairment is primarily caused by physical frailty and manifests as reversible cognitive frailty [1]. Cognitive frailty not only affects the quality of life of older adults but also significantly increases the risk of severe adverse outcomes such as dementia and disability [2]. With the accelerating aging of the global population, cognitive frailty has become an important issue in the public health domain. Estimates from Meta-analyses indicate that the global overall prevalence of cognitive frailty ranges from 9.0% to 16% [3]. Meanwhile, a cross-sectional study revealed that the prevalence of cognitive frailty among older adults in Thai communities is 28.72% [4].

ERGO, a naturally occurring antioxidant, is an unusual thio-histidine betaine amino acid that has gradually revealed powerful antioxidant, anti-inflammatory, and cytoprotective properties since it was first isolated from fungi (*Claviceps purpurea*) in 1909 [5]. Due to its unique physiological activity and broad safety application prospects, ERGO has garnered significant attention in recent years in the study of various chronic diseases. This article aims to review the research progress of ERGO in the treatment of cognitive frailty and explore its potential application value.

## 2. Biological functions of ERGO

### 2.1. Chemical structure and sources

**Figure 1.**
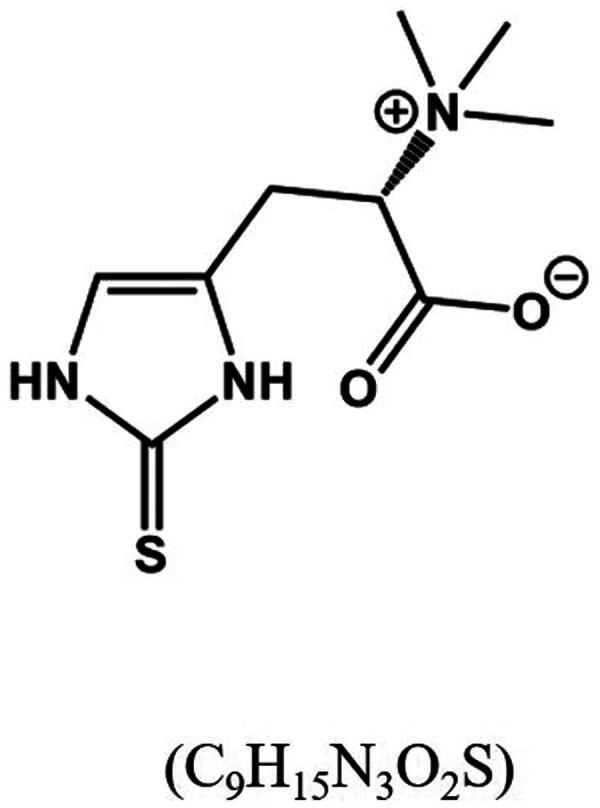
ERGO chemical structure (C_9_H_15_N_3_O_2_S).

The chemical name of ERGO is 2-mercaptohistidine trimethylbetaine (C_9_H_15_N_3_O_2_S, see Figure 1), which exhibits strong thermal and acid-base stability. ERGO is mainly synthesized by actinomycetes, cyanobacteria, and certain fungi (for instance, *Streptomyces* spp., *Burkholderia* spp. and *Mycobacterium spp*). Although mammals cannot synthesize ERGO themselves, they can acquire it through their diet, especially from foods such as mushrooms, beans, and garlic [6,7]. The distribution of ERGO in the human body is selective, and the novel organic cation transporter 1 (OCTN1) is its primary transporter, widely distributed in various tissues including the brain, digestive tract, liver, heart, spleen and kidney [8]. Research has shown that ERGO can enter the central nervous system (CNS) through the blood–brain barrier (BBB) and be detected in human cerebrospinal fluid and postmortem brain tissue [9,10].

### 2.2. Antioxidant and anti-inflammatory effects

ERGO is a direct scavenger of reactive oxygen species, effectively neutralizing free radicals and reducing oxidative stress damage. Additionally, ERGO can indirectly exert antioxidant effects by upregulating proteins responsible for the oxidative defense pathway [11]. Besides its antioxidant function, ERGO also possesses significant anti-inflammatory properties, capable of reducing the expression of various inflammatory factors and inhibiting inflammatory responses [12]. Research has demonstrated that ERGO exhibits significant protective effects against 7-ketocholesterol (7KC)-induced mitochondrial damage in human brain endothelial cells, as evidenced by improved cell viability, reduced intracellular calcium levels, decreased reactive oxygen species, and modulated expression of inflammatory markers [13]. ERGO can directly inhibit the transcriptional activity of the NF-κB signaling pathway, reduce the production of pro-inflammatory cytokines such as TNF-α and IL-6, and thus suppress neuroinflammatory responses [14]. Chronic activation and region-specific overreaction of microglia, especially in the white matter area, are important driving factors for cognitive fragility [15]. Wild type microglia cells exposed to ERGO showed a significant reduction in cell hypertrophy induced by LPS stimulation, as well as a decrease in intracellular reactive oxygen species, indicating that OCTN1 mediated ERGO uptake may inhibit microglial activation induced cell hypertrophy by suppressing ROS production [16].

### 2.3. Potential therapeutic for COVID-19-related neurological complications

With the outbreak of the COVID-19 pandemic, symptoms of cognitive frailty may persist following long-term infection with the novel coronavirus, thereby affecting patients’ quality of life and functional abilities [17]. In numerous studies on the severity of coronavirus infection, hypoxia-induced respiratory distress, cytokine storm-induced central nervous system (CNS) inflammatory damage, and hypercoagulability leading to cerebral venous thrombosis or stroke have all been identified as major risk factors for mortality or severe symptoms [18]. Since ERGO has various cell-protective properties, Cheah et al. proposed ERGO can be used as a therapeutic drug to reduce the severity and mortality of COVID-19, especially in the elderly and people with potential health conditions [19]. ERGO has shown promise in mitigating neuroinflammation and oxidative stress in preclinical models of cerebrovascular injury. For example, it reduces 7-ketocholesterol-induced endothelial damage, suppresses pro-inflammatory cytokines, and preserves tight junction protein integrity in a brain microvascular endothelial hCMEC/D3 cell line [20]. While these mechanisms align with pathways implicated in neuroinflammation, direct evidence linking ERGO to COVID-19-related CNS pathology remains absent. Further studies are needed to explore its therapeutic potential in SARS-CoV-2-induced neuroimmune dysregulation.

### 2.4. Other biological functions

ERGO exhibits several other biological functions, including immunomodulation, cyto-protection, promotion of neural cell differentiation, and anti-aging effects. Studies have shown that ERGO can slow down high glucose-induced endothelial cell senescence by activating the SIRT1 and SIRT6 signaling pathways [21]. It can also alleviate ultraviolet-induced fibroblast senescence through activation of the Nrf2/HO-1 pathway and HSP70 in keratinocytes [22]. Furthermore, ERGO can reduce the telomere shortening rate of primary human fibroblasts, exerting an antiaging effect [23]. Simultaneously, ERGO, due to its unique structure featuring a sulfur-substituted imidazole ring [24], inhibits tyrosinase activity, thereby exhibiting a mechanism of action that suppresses melanin production or removes melanin [25]. OCTN1 in neural progenitor cells transports ERGO, inhibiting proliferation and promoting neuronal differentiation *via* Math1 up-regulation. ERGO, unlike edaravone and ascorbic acid, affected differentiation. OCTN1 knockdown reversed these effects, suggesting a unique mechanism beyond antioxidant action [26]. This ability may be beneficial for repairing neuronal damage caused by coronavirus infiltration and injury to brain neurons. Through multitarget synergistic actions, ERGO mitigates the aging process by acting on antioxidant, anti-inflammatory, epigenetic regulation, and mitochondrial function maintenance pathways. Its mechanisms involve key biological processes such as the Sirtuins pathways, KEAP1–NRF2 axis, suppression of cellular senescence, and neuroprotection [27]. Clinical studies have demonstrated that ergothioneine supplementation improves memory and attention in older patients with mild cognitive impairment (MCI) [28].

## 3. Multifactorial nature of cognitive frailty

Cognitive Frailty is associated with a heightened risk of falls, fractures, disability, hospitalization, mortality, and dementia, while also reducing health-related quality of life and increasing social isolation. Its occurrence and development are not driven by a single factor but are mediated by a complex network of systemic aging processes, pathological changes at the molecular and cellular levels, organ system interaction disorders, and psychosocial factors.

### 3.1. Systemic aging and accelerated aging

Aging itself is the biological foundation of cognitive frailty. ‘Age acceleration’—a metric quantifying the deviation between biological age and chronological age—further characterizes the heterogeneity of individual aging rates [29,30]. Concurrently, epigenetic clocks such as Horvath clock and GrimAge clock utilize DNA methylation profiles to predict biological age with precision. Accelerated epigenetic aging is significantly associated with hippocampal volume atrophy and decline in working memory [31], suggesting that dysregulation of epigenetic regulatory networks may initiate neurodegenerative processes during the early stages of cognitive frailty. Additionally, the concept of cognitive reserve refers to the ‘buffering capacity’ accumulated by individuals through education, cognitive training, social activities, etc., which may delay or alleviate age-related cognitive decline [32]. Executive dysfunction is usually a symptom of a disease characterized by cognitive impairment. Higher frailty is associated with executive dysfunction in middle- and older-aged adults, with cardiovascular health partially mediating this relationship (∼10% mediation, except in middle-aged females) [33].

### 3.2. Vascular–neural unit injury

Degenerative changes in the cerebral vascular system are a critical factor in cognitive frailty [34]. Cerebral small vessel disease, the most prevalent cerebrovascular pathology, disrupts the myelin integrity of white matter through white matter hyperintensities (WMH), lacunar infarcts, and microbleeds, interfering with information transmission along white matter tracts [35]; Simultaneously, increased blood-brain barrier permeability facilitates the infiltration of plasma toxic factors into brain parenchyma, activating inflammatory responses in microglia and astrocytes [36]. Moreover, atrial fibrillation (AF) increases the risk of vascular cognitive impairment (VCID) and dementia through mechanisms such as thromboembolism, cerebral hypoperfusion, inflammation, and microbleeds, COVID-19 may exacerbate the undiagnosed rate of AF, leading to delayed detection of cognitive impairment [37]. systemic cardiovascular aging exacerbates spatiotemporal heterogeneity in cerebral blood flow (CBF) by lowering cerebral perfusion pressure (CPP), disproportionately affecting regions highly dependent on sustained perfusion, such as the prefrontal cortex and hippocampus, ultimately leading to declines in neuronal plasticity and survival [38]. Canonical correlation analysis (CCA) revealed the significant impact of neurovascular unit (NVU) dysfunction on cognitive performance in patients with cerebral small vessel disease (cSVD), underscoring NVU as an integrated functional unit governing cognitive outcomes [39].

### 3.3. Chronic inflammation and metabolic disorders

‘Inflammaging’, a hallmark of chronic low-grade inflammation during aging, is characterized by age-dependent elevation of GDF15, a senescence-associated biomarker positively correlated with inflammatory markers such as hypersensitive C-reactive protein, hsCRP. Inflammation serves as a shared pathological mechanism underlying cognitive frailty and depression, with GDF15 potentially modulating this process by regulating inflammatory responses, such as suppressing macrophage activation [40]. As a critical regulator of synaptic plasticity, impaired insulin signaling directly weakens learning and memory functions. The metabolic dysfunction and decreased metabolic flexibility caused by aging are important driving factors for physical weakness [41,42]. Phosphorus magnetic resonance spectroscopy (^31^P MRS) noninvasively measures ratios of high-energy phosphates and phospholipid metabolism in the brain, reflecting energy reserves, consumption, and demand. Research utilizing ^31^P MRS has elucidated dynamic associations between cerebral energy metabolism and cognitive function in older adults, highlighting mitochondrial dysfunction as a core driver of cognitive decline [43].

### 3.4. Neurodegenerative changes and functional network remodeling

Cognitive frailty often culminates in the accumulation of neurodegenerative pathology, with amyloid-beta (Aβ) deposition and hyperphosphorylated tau protein serving as core features of Alzheimer’s disease (AD). However, the onset of these pathological hallmarks in cognitive frailty can precede clinical symptoms by years to decades [44]. Concurrently, dynamic reconfiguration of functional connectivity (FC) reflects the compensatory and decompensatory processes of brain networks in response to pathological damage. The default mode network (DMN), a primary resting-state network, exhibits weakened internal connectivity, such as reduced FC between the posterior cingulate cortex and hippocampus, which correlates closely with episodic memory impairment. Abnormal cross-network connections between the executive control network (ECN) and frontoparietal networks directly impair working memory and decision-making abilities [45]. Utilizing the ADNI(Alzheimer’s Disease Neuroimaging Initiative) database, researchers validated the synergistic role of molecular (DNA methylation age, MA), phenotypic (brain age, BA), and functional (frailty index, FI) biomarkers in dementia prediction. The combined MA–BA model demonstrated superior predictive accuracy for distinguishing cognitively normal individuals (CN) from dementia patients and mild cognitive impairment (MCI) from dementia stages, with AUC values of 0.82 and 0.83, respectively [46]. These findings underscore that multimodal data integration including biological features and clinical manifestations enhances the precision of cognitive disease prediction.

### 3.5. Psychosocial stress

Psychosocial factors such as chronic stress, depression, and social isolation exert bidirectional regulatory effects on physiological aging processes through the ‘brain–peripheral organ’ axis, becoming a critical upstream risk factor for cognitive frailty. The complex association between a history of depression and Alzheimer’s disease (AD), cerebrovascular disease, and neurodegenerative biomarkers provides important pathological evidence for cognitive vulnerability [47]. Social support from family, friends, or community networks can mitigate the negative impact of psychosocial stress on cognitive frailty. For example, a study of older adults demonstrated that social support indirectly reduced cognitive frailty by alleviating psychological distress such as anxiety, depression [48]. Genetic vulnerability and neurodevelopmental abnormalities, such as amygdala dysfunction, may amplify the detrimental effects of psychosocial stress on cognitive function [49]. Besides, single sensory impairments such as vision, hearing, smell, and touch are all associated with cognitive decline [50,51].

Above all, the essence of cognitive fragility is a ‘domino network’ constructed by systemic aging, molecular pathology, organ dysfunction, and psychosocial factors. Systemic aging provides a cumulative effect in the time dimension, vascular and metabolic abnormalities disrupt the homeostasis of the brain microenvironment, neurodegeneration and functional network reconstruction mark the depletion of brain reserves, and psychosocial stress acts as an upstream driving factor, amplifying other pathological processes through neuroendocrine and immune axes. The complexity of cognitive frailty—rooted in multisystem interactions, temporal ambiguity, and individual heterogeneity—poses significant challenges to traditional intervention models. However, it also creates a unique opportunity for ERGO-based approaches, which leverage environmental, behavioral, and social factors to address cognitive fragility’s interconnected pathology. By integrating precision biomarkers, targeting multiple pathways synergistically, and prioritizing long-term sustainability, ERGO interventions can serve as a foundational component of the broader therapeutic landscape—complementing pharmacotherapies, preventing cognitive decline, and improving quality of life for older adults at risk of or living with cognitive fragility.

## 4. ERGO and cognitive frailty

### 4.1. Blood ERGO as cognitive health indicator

Multiple studies have indicated a decreasing trend in plasma ERGO levels across various diseases, suggesting a potential close correlation between reduced ERGO levels and the development of these diseases. Notably, plasma ERGO levels have been found to significantly decline in patients with cognitive decline and dementia [28]. Research into the changes of ERGO in patients with cognitive frailty has revealed that plasma ERGO levels gradually decrease with age, and elderly individuals with impaired cognitive function have significantly lower plasma ERGO levels compared to cognitively normal elders [52]. Through an analysis of plasma metabolic markers in dementia patients, Teruya et al. discovered a notable reduction in plasma ERGO and its synthesis-related compounds [53]. Low blood ERGO levels are linked to cognitive impairment and cerebrovascular disease. In a study of 470 elderly Singaporeans, lower plasma ERGO levels were associated with poorer baseline cognitive performance and faster decline in multiple cognitive domains. These findings support ERGO as a potential biomarker for accelerated cognitive and functional decline, also suggesting therapeutic and preventative measures [54]. A cohort study conducted on Japanese community residents showed that serum ergothioneine concentrations peaked in men during their 70s and in women during their 60s, with concentrations being higher in women than in men [55]. In a study conducted in Korea, ergothioneine concentrations in blood samples stored at room temperature for up to 4 h were significantly higher in the elderly group compared to the younger group [56].

### 4.2. ERGO’s importance role in metabolites

Metabolomic studies refer to scientific investigations that systematically analyze and identify the complete set of metabolites (small molecule biochemicals) present within a biological sample, such as blood, urine, tissue, or cells. Metabolomic studies have also implicated ERGO in cognitive function. For instance, in a study examining the blood metabolome of cognitive function and brain health in middle-aged adults, ERGO exhibited the largest effect on general cognition (G-factor) among the 14 metabolites significantly associated with cognitive performance [57]. Another study explored the effectiveness and safety of the dietary compound ERGO in delaying cognitive decline in elderly individuals, a double-blind, randomized, placebo-controlled study found that ERGO intake did not alter clinical safety indicators, validating its safety. The ERGO group performed better than the placebo group in terms of learning ability assessment and stable levels of neurofilaments, indicating that ERGO may help improve memory and learning abilities, slow down neuronal damage, and have the potential to delay cognitive decline in the elderly [58]. The brain is one of the organs with the highest energy consumption, and its neuronal activity is highly dependent on ATP produced by mitochondrial metabolism [59]. MPST is implicated in one-carbon unit metabolism, and the metabolites of this pathway, for example S-adenosylmethionine (SAM) are critical for mitochondrial function. Changes in SAM levels directly influence the stability of mitochondrial complex I. Research indicates that ERGO promotes mitochondrial respiration and exercise performance by directly activating 3-mercaptopyruvate sulfurtransferase (MPST) [60]. Recent research has found that ERGO enhances anaerobic energy metabolism in human gut bacteria. Intestinal bacteria from different phyla metabolize ERGO to produce energy under anaerobic conditions, which may be achieved through metabolic cross feeding [61].

### 4.3. ERGO as pathogen virulence factor

Beneficial bacteria are associated with improvements in memory and attention, while harmful bacteria are associated with mild cognitive impairment. ERGO can synthesize in actinomycetes, cyanobacteria, methylobacteria, and fungi, differs from glutathione and mycothiol by being a thione at physiological pH, enhancing microbial survival under stress. It maintains bioenergetic homeostasis, protects against oxidative stress, and augments drug resistance in Mycobacterium tuberculosis, enhancing virulence. Aspergillus fumigatus also produces ergothioneine, critical for conidial health and fungal resistance [62]. Meanwhile, some pathogens that cannot synthesize ERGO, such as E. coli [63] and the oral pathogen Treponema denticola [64], can accumulate and utilize ERGO from the environment, facilitating the exploitation by opportunistic pathogens and thereby enhancing their pathogenicity. However, the molecular mechanisms of ERGO ‘s functions in microbial physiology and pathogenesis are unclear, especially the impact of microbial synthesized ERGO on cognitive Frailty is not clear, necessitating further research to advance our understanding.

### 4.4. Potential public health impact of ERGO

In a cohort study of older community-dwelling adults in Australia, it was observed that serum ERGO concentrations decreased while asymmetric dimethyl-l-arginine concentrations increased with increasing psychological distress. After adjusting for age and sex, only ERGO remained independently associated with psychological distress, with each unit increase in ERGO reducing the odds of its occurrence by approximately 68%. This trend suggests a potential link between ERGO and mental health. Given its dietary origin, integrating ERGO offers therapeutic opportunities that warrant further investigation in intervention studies [65].

## 5. Mechanism research progress of ERGO in the treatment of frailty and cognitive impairment

### 5.1. ERGO improves cognitive function and neuronal health

Evidence from animal studies suggests that ERGO significantly improves frailty and cognitive impairment (Table 1). Studies have shown that ERGO-induced neuronal differentiation is partly mediated by the phosphorylation of S6K1 at Thr389 and the subsequent activation of TrkB signaling induced by NT5. Repeated oral administration of ERGO or food extract tablets containing ERGO has been found to enhance memory function in mice [66,67]. ERGO counteracts cisplatin-induced neurotoxicity, significantly restoring cisplatin-induced learning and memory deficits in mice. It reduces neuronal damage by inhibiting oxidative stress and restoring acetylcholinesterase activity, maintained glutathione/glutathione disulfide ratio [68]. In an Aβ-induced rat model, ERGO pretreatment can alleviate apoptosis and cell death, primarily by inhibiting the formation of peroxynitrite and the nitration of protein tyrosine residues [69].

**Table 1.** Summarizing the mechanisms of ERGO in treating cognitive frailty.

Research area	Key targets/pathways	Specific effects	Relevant citations (literature references)
Antioxidant effect	Nrf2/HO-1,KEAP1-NRF2 pathway, Antioxidant defense pathways, ROS (•O₂¯, H₂O₂, ONOO¯).	1. Directly scavenges reactive oxygen species (ROS). 2. Upregulates antioxidant proteins (SOD, CAT, GPx). 3. Reduces malondialdehyde (MDA) levels.	[11,27,66,68,69,73,75]
Anti-inflammatory effect	NF-κB signaling pathway, Pro-inflammatory cytokines (e.g. TNF-α, IL-6)	1. Inhibits NF-κB activation and downstream cytokine production. 2. Suppresses microglial activation and neuroinflammation.	[12–16,20,79]
Neuroprotective effect	Neuronal apoptosis pathways, synaptic integrity, BDNF-TrkB signaling	1. Reduces Aβ-induced oxidative damage and tau hyperphosphorylation. 2. Promotes neuronal survival and synaptic plasticity.	[65,67–69,73,76]
Improvement of cognitive function	Cognitive-related pathways, Aβ deposition, cerebral blood flow	1. Enhances memory and learning abilities. 2. Reduces amyloid plaque deposition and brain atrophy.	[65,73,76,78]
As a biomarker	Plasma ERGO levels, metabolic markers (e.g. GDF15, hsCRP)	1. Correlates with cognitive decline severity. 2. Predicts progression from mild cognitive impairment (MCI) to dementia.	[28,52–54,77]
Metabolic regulation	SIRT1/SIRT6, Nrf2/HO-1, mitochondrial function	1. Activates SIRT1/6 to delay cellular senescence. 2. Maintains mitochondrial membrane potential and ATP synthesis.	[21,60,72]
Vascular protection	Blood-brain barrier (BBB) integrity, tight junction proteins (Claudin-5, ZO-1)	1. Preserves BBB integrity and reduces endothelial permeability. 2. Improves cerebral microcirculation and glucose metabolism.	[9,10,36]
Anti-aging effects	Telomerase activity, SIRT6, Nrf2/HO-1 pathway	1. Inhibits telomere shortening under oxidative stress. 2. Reducing MDA content and enhancing T-SOD activity.	[21,23,72,73]

Using a cohort of *n* = 176 healthy elders from the Harvard Aging Brain Study (HABS), research indicates that global structural-functional connectivity coupling (global SFC) exhibits a negative correlation with cognitive performance, such that lower SFC is associated with worse cognitive function [70]. Notably, low plasma ERGO levels are significantly linked to cognitive decline, particularly in non-demented elderly individuals. This association may be mediated by cerebrovascular lesions such as white matter hyperintensities (WMH) and brain atrophy. These findings suggest that ERGO may indirectly preserve cognitive function by maintaining cerebrovascular health and stabilizing SFC [54]. Neurodegenerative diseases are often accompanied by a decrease in synaptic plasticity. It is still uncertain whether ERGO may indirectly maintain synaptic plasticity by delaying neuronal damage, and further research is also warranted [71].

### 5.2. ERGO attenuates oxidative stress and enhances neuroprotection

In a d-galactose-induced aging mouse model, ERGO intervention significantly improved recognition memory and neuronal survival, while enhancing levels of cAMP and BDNF in the brain. Additionally, ERGO was able to attenuate oxidative stress by reducing MDA content and enhancing T-SOD activity *via* the Nrf2/HO-1 pathway, further supporting its neuroprotective effects [72]. Oral ERGO enhances learning and memory in mice with hippocampal Aβ, reduces amyloid plaques, oxidative stress, and brain lipid peroxidation, improves glucose metabolism, and repairs mitochondrial dysfunction, and alleviating nervous system damage [73].

### 5.3. ERGO optimizes glucose metabolism and energy supply in neurodegenerative models

**Figure 2.**
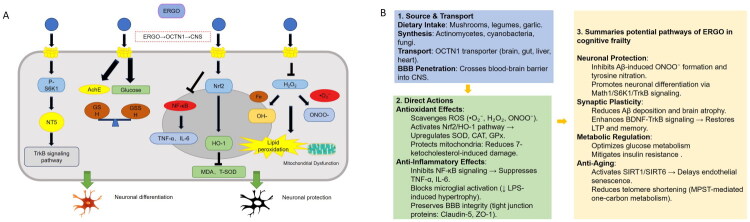
(A) ERGO exerts its neuroprotective effects by influencing neuronal differentiation and antioxidant capacity, thereby consistently mitigating cognitive frailty. (B) Sources, transport, actions and hypothesized pathways of ERGO in cognitive frailty.

In a study, ERGO treatment significantly improved glucose metabolism in the 5XFAD mouse model. ERGO effectively alleviated oxidative stress-induced damage to neurons, and further optimized glucose metabolism, thereby restoring energy supply to brain cells. This mechanism plays a crucial role in maintaining and enhancing cognitive function [74]. In high-risk populations for type 2 diabetes (T2D), long-term consumption of foods rich in ERGO, such as white mushrooms, significantly enhances antioxidant capacity, improves inflammatory responses, and reduces oxidative stress markers associated with diabetes. Furthermore, these findings indicate that ergothioneine may not only influence metabolic changes in cognitive frailty but also potentially modulate the pathological processes of T2D through its antioxidant effects [75]. Also, intervention with *Pleurotus eryngii*, which is rich in ERGO, in an Aβ-induced mouse model revealed improvements in memory and learning abilities, reduction in Aβ plaque deposition, and alleviation of brain atrophy [76]. Overall, ERGO demonstrates potential therapeutic benefits in addressing aspects of frailty and cognitive impairment, with its effects likely mediated through multiple mechanisms, although further clinical validation is necessary (Figure 2).

## 6. Current status of clinical research on ERGO

Emerging evidence from preclinical and clinical studies underscores its safety and bioactivity, prompting multiple ongoing trials to validate its efficacy in diverse pathological contexts (Supplemental Table 1). The National University Hospital, Singapore (NCT03641404) aims to evaluate the effectiveness of ERGO in delaying cognitive decline among patients with mild cognitive impairment, with results indicating that individuals treated with ERGO exhibited improved performance in learning ability assessments [57]. The Midwest Center for Metabolic and Cardiovascular Research’s (NCT04556032) investigation into ERGO’s effects on cognition, mood, and sleep in healthy adults has been terminated. Meanwhile, Natural Immune Systems Inc.’s (NCT05042674) study on ERGO’s rapid antioxidant protection and immune modulation is listed as unknown. Despite variations in progress and outcomes, they provide valuable data and insights for developing this dietary supplement’s clinical value.

Although there are currently no much clinical trials directly investigating the intervention of ERGO in cognitive frailty, epidemiological studies have shown a close correlation between mushroom intake and improved cognitive function. Mushrooms are the primary dietary source of ERGO. A study focusing on American elderly individuals found a significant association between increased mushroom intake and improved cognitive abilities [77]. This discovery provides indirect evidence for the application of ERGO in the prevention and treatment of cognitive frailty.

## 7. Future Research directions and challenges

### 7.1. Improving animal model research

Currently, there is a lack of suitable animal models for cognitive frailty to comprehensively evaluate the therapeutic effects of ERGO. Future efforts should focus on developing animal models that are more closely aligned with the pathophysiological mechanisms of human cognitive frailty, to further investigate the mechanism and effectiveness of ERGO in cognitive frailty. Also, current animal models of cognitive frailty lack translational relevance to human pathophysiology. More studies should develop aged rodent models with comorbidities to replicate clinical scenarios. We propose longitudinal interventions combining ERGO supplementation with *in vivo* imaging to dynamically assess cerebral blood flow and microvascular integrity. Spatial transcriptomics can further map ERGO’s neuroprotective signatures across cortical and hippocampal regions.

### 7.2. Conducting clinical trials

Given the promising results demonstrated by ERGO in animal experiments, clinical trials should be actively pursued in the future to investigate the safety and efficacy of ERGO in intervention for cognitive frailty in humans. In particular, studies in naturally aging animal models will provide important evidence for the application of ERGO in the elderly population. While existing trials (e.g. NCT03641404) have confirmed the safety of ERGO, evidence supporting its efficacy remains limited. To address this gap, longitudinal randomized controlled trials (RCTs) should integrate comprehensive neurovascular endpoints, including standardized cognitive assessments alongside serum biomarker panels including ERGO levels, inflammatory markers such as IL-6 and TNF-α, oxidative stress indicators like MDA. Advanced neuroimaging modalities—such as arterial spin labeling MRI (ASL-MRI) for cerebral perfusion evaluation and diffusion tensor imaging (DTI) for white matter integrity assessment—should also be incorporated. Subgroup analyses focusing on populations with diabetes or hypertension are warranted to explore potential metabolic modulation effects. Preclinical studies often utilize high-dose ERGO supplementation to demonstrate efficacy, achieving comparable systemic concentrations in humans may be hindered by limited intestinal absorption and rapid metabolic clearance, thereby necessitating dose optimization studies to reconcile these disparities. Notably, a minimum 3-year follow-up period is essential to capture longitudinal cognitive trajectories and determine the sustained impact of ERGO.

### 7.3. Exploring combination therapy

The pathogenesis of cognitive frailty is complex, and a single drug treatment may not be able to fully address it. Future research can explore the combined application of ERGO with other interventions such as exercise, nutritional support, etc., aiming to achieve better therapeutic effects. ERGO interventions do not replace targeted pharmacotherapies but rather complement them by addressing the ‘context’ in which pathological processes unfold. For example, in Alzheimer’s disease (AD), anti-amyloid therapies may clear plaques [78], but ERGO interventions can enhance synaptic resilience and reduce inflammation, thereby improving the functional impact of plaque clearance. In vascular cognitive impairment, blood pressure-lowering medications may reduce CSVD progression [79], but ERGO’s focus on metabolic can further improve endothelial function and cerebral blood flow.

### 7.4. Emerging technologies for mechanistic dissection

Traditional assays lack spatial resolution to delineate ERGO’s CNS effects. Multi-omics approaches—including single-nucleus RNA sequencing of postmortem brain tissues and proteomic profiling of cerebrospinal fluid—can identify ERGO-responsive pathways. Machine learning models integrating metabolomic, imaging, and clinical data may predict ERGO responsiveness.

## 8. Conclusion

While ERGO demonstrates promising antioxidant, anti-inflammatory, and neuroprotective properties in preclinical models, the current evidence base for its therapeutic efficacy in cognitive frailty remains preliminary. Although observational studies correlate low plasma ERGO levels with cognitive decline and dementia risk, randomized controlled trials (RCTs) specifically targeting cognitive frailty are scarce. Existing clinical data, such as the Singapore-based NCT03641404 trial, suggest potential benefits in mild cognitive impairment populations, but these findings require replication in larger, longer-duration studies with standardized cognitive outcome measures. Moreover, mechanistic insights from animal models must be validated in human cohorts. The interplay between ERGO’s antioxidant effects and key pathological drivers of cognitive frailty—such as neuroinflammation, vascular dysfunction, and tauopathy—also warrants deeper investigation. In summary, while ERGO holds theoretical promise for cognitive frailty, overstated conclusions risk misguiding clinical translation. A balanced discussion of current limitations—coupled with strategic calls for mechanistic and clinical research—is essential to advance this field.

## Supplementary Material

supplement table 1.docx

## Data Availability

No new data were generated or analyzed in this study. The data used in this work are based on previously published sources.

## Abbreviations

ERGO: Ergothioneine
OCTN1: Organic cation transporter 1
CNS: Central nervous system
T2D: Type 2 diabetes
ROS: Reactive oxygen species
NF-κB: Nuclear factor kappa-B
BDNF: Brain-derived neurotrophic factor
MCI: Mild cognitive impairment
ADNI: Alzheimer’s disease neuroimaging initiative
SIRT1/SIRT6: Sirtuin 1/6
Complex I: Mitochondrial respiratory chain complex I
ADMA: Asymmetric dimethyl-l-arginine
GDF15: Growth differentiation factor 15
hsCRP: Hypersensitive C-reactive protein
