# Supplementary material for: Advances and prospects of ergothioneine in the treatment of cognitive frailty
Source: Ann Med. 2025 Sep 7;57(1):2555742. doi: 10.1080/07853890.2025.2555742 (PMC12416020; doi:10.1080/07853890.2025.2555742)
Supplement: supplement table 1.docx [file IANN_A_2555742_SM9757.docx]

Supplement Table 1 Registration of clinical studies on ERGO

| NCT Number | Study Title | Study Status | Conditions | Interventions | | Sponsor | Collaborators | Study Type |
| --- | --- | --- | --- | --- | --- | --- | --- | --- |
| NCT03641404 | Investigating the Efficacy of Ergothioneine to Delay Cognitive Decline | UNKNOWN | Mild Cognitive Impairment | DIETARY_SUPPLEMENT: ergothioneine  \|DIETARY_SUPPLEMENT: placebo | | National University Hospital,  Singapore | National University of Singapore\|National University Health System, Singapore | INTERVENTIONAL |
| NCT04556032 | Effects of Ergothioneine on Cognition, Mood, and Sleep in Healthy Adult Men and Women | TERMINATED | Healthy | DIETARY_SUPPLEMENT: L-Ergothioneine 10 mg/d  \|DIETARY_SUPPLEMENT: L-Ergothioneine 25 mg/d  \|OTHER: Placebo | | Midwest Center for Metabolic and Cardiovascular Research | Blue California Company | INTERVENTIONAL |
| NCT05042674 | Clinical Study on Rapid Antioxidant Protection and Immune Modulating Effects. | UNKNOWN | Pharmacokinetics | DIETARY_SUPPLEMENT: Placebo\|  DIETARY_SUPPLEMENT: Ergothioneine, 25 miligrams\|  DIETARY_SUPPLEMENT: Ergothioneine, 25 miligrams, daily for 1 week | Natural Immune Systems Inc | |  | INTERVENTIONAL |
